# Seroprevalence and SARS-CoV-2 antibodies distribution in Portugal following mass vaccination campaign

**DOI:** 10.1093/eurpub/ckac131.411

**Published:** 2022-10-25

**Authors:** I Kislaya, P Gonçalves, V Gaio, R Matos, M Barreto, A Melo, R Guiomar, A P Rodrigues, PT ISNCOVID-19 group

**Affiliations:** 1 Department of Epidemiology, National Institute of Health Doutor Ricardo Jorge, Lisbon, Portugal; 2 Department of Infectious Diseases, National Institute of Health Doutor Ricardo Jorge, Lisbon, Portugal

## Abstract

**Introduction:**

Information on post-infection and vaccine-induced SARS-CoV-2 seroprevalence is important for public health policies. A 3rd wave of National Serological Survey (ISN3COVID-19) was conducted to measure SARS-CoV-2 seroprevalence and characterize specific antibodies distribution in Portuguese population in September - November 2021, following a mass vaccination campaign.

**Methods:**

ISN3COVID-19 was a cross-sectional epidemiological study that collected serum samples and questionnaire data on a sample of Portuguese residents aged 1 year or older (n = 4545). SARS-CoV-2 IgG anti-nucleoprotein and anti-spike antibody levels were measured using Abbott Chemiluminescent Microparticle Immunoassays. Seroprevalence was estimated for the overall sample and stratified by age group, sex, region and self-reported chronic conditions. Medians and respective 95% confidence intervals (95%CI) were used to describe the distribution of SARS-CoV-2 antibodies in specific population subgroups.

**Results:**

The overall seroprevalence of SARS-CoV-2 (post-infection or vaccine-induced) was 86.4% (95%CI: 85.2 to 87.6%), post-infection seroprevalence was 7.5% (95%CI: 6.6 to 8.5). Higher seroprevalence was observed among 50-59 years-old (96.5%), women (88.3%), and those with two or more self-reported chronic conditions (90.8%). Higher IgG (anti-Spike) levels were estimated for individuals vaccinated with the booster dose (median=12601.3 AU/ml; 95%CI: 4127.5 to 19089.1) and for those vaccinated with two doses of Spikevax® vaccine (median=7012.7 AU/ml, 95%CI: 5568.8 to 8456.6).

**Conclusions:**

The SARS-CoV-2 seroprevalence was high and consistent with vaccine coverage in Portugal. Seropositivity was associated with sex, age and previous chronic conditions. The anti-SARS-CoV-2 anti-spike IgG levels varied according to vaccine brand and number of doses. These results show that monitoring seroprevalence and SARS-CoV-2 antibody distribution is of paramount importance to guide public health policies.

**Key messages:**

• Significant increase in SARS-CoV-2 seroprevalence following the mass vaccination campaign consistent with the high vaccine coverage achieved in Portugal.

• Continuous monitoring of the population‐level IgG response after vaccination remains important to guide further public health measures.

